# A Web-Based Intervention for Relatives of People Experiencing Psychosis or Bipolar Disorder: Design Study Using a User-Centered Approach

**DOI:** 10.2196/11473

**Published:** 2018-12-07

**Authors:** Mahsa Honary, Naomi Ruth Fisher, Roisin McNaney, Fiona Lobban

**Affiliations:** 1 School of Computing and Communications Lancaster University Lancaster United Kingdom; 2 Spectrum Center for Mental Health Research Division of Health Research Lancaster University Lancaster United Kingdom

**Keywords:** mental health, Web-based intervention, user-centered design, caregivers, bipolar disorder, psychosis

## Abstract

**Background:**

Relatives of people experiencing bipolar mood episodes or psychosis face a multitude of challenges (eg, social isolation, limited coping strategies, and issues with maintaining relationships). Despite this, there is limited informational and emotional support for people who find themselves in supporting or caring roles. Digital technologies provide us with an opportunity to offer accessible tools, which can be used flexibly to provide evidence-based information and support, allowing relatives to build their understanding of mental health problems and learn from others who have similar experiences. However, to design tools that are useful to relatives, we first need to understand their needs.

**Objective:**

The aim of this study was to use a user-centered design approach to develop an accessible Web-based intervention, based on the Relatives Education And Coping Toolkit (REACT) booklet, to support the informational and emotional needs of relatives of people experiencing psychosis or bipolar disorder.

**Methods:**

We engaged relatives of people with experiences of bipolar disorder or psychosis in workshops to identify their needs and design requirements for developing a Web-based version of a paper-based toolkit. We used a 2-phase qualitative approach to explore relatives’ views on content, design, and functionalities, which are considered to be engaging and useful in a Web-based intervention. In phase 1, we consulted 24 relatives in 2 workshops to better understand their existing support infrastructure, their barriers for accessing support, unmet needs, and relatives’ views on online support. On the basis of the results of these workshops, we developed a set of design considerations to be explored in a smaller workshop. Workshop 3 then involved working with 2 digitally literate relatives to design a usable and acceptable interface for our Web-based toolkit. Finally, in phase 2, we conducted a heuristic evaluation to assess the usability of the toolkit.

**Results:**

Our findings indicated that relatives require technologies that (1) they can place their trust in, particularly when discussing a highly sensitive topic, (2) enable learning from the lived experiences of others while retaining confidentiality, and (3) they can work through at their own pace in a personalized manner.

**Conclusions:**

Our study highlights the need for providing a trustworthy, supportive tool where relatives can engage with people who have similar experiences to their own. Our heuristic evaluation showed promise in terms of perceived usability of the REACT Web-based intervention. Through this work, we emphasize the need to involve stakeholders with various characteristics, including users with limited computer literacy or experience in online support.

## Introduction

### Background

Psychosis is an umbrella term that covers many different conditions, the common feature of which is a loss of touch with reality. The most common ways this manifests in are believing things that are generally accepted to be untrue by other people (often called delusions); not being able to think straight, thus sounding very muddled and confused (often called thought disorder); experiencing things that are not actually happening, for example, hearing or seeing things that other people cannot (often called hallucinations).

Along with the presence of these unusual experiences, many people with psychosis also report a loss of valued experiences, most notably, pleasure in everyday activities (anhedonia) and loss of motivation (apathy). These losses are sometimes referred to as *negative symptoms* and are particularly challenging for relatives: not least because they are hard to differentiate from normal *teenage angst*, side effects of medication, or depression. It is difficult to report exact figures on the number of people who will experience psychosis as many may never have contact with mental health services. However, most recent estimates include worldwide incidence at approximately 1 in 13 people (7.7%; [1]) and up to 10% in the United Kingdom [2]. Only a fraction of these people will come into contact with mental health services and receive a diagnosis of a mental health condition. In general, these are likely to be people for whom these experiences are particularly distressing or cause significant changes in behavior.

Bipolar disorder (BD) is the third most common mental health cause of disability globally [3], affecting 1% to 4.5% of adults [4] and costing the English economy £5.2 billion annually, largely because of inadequate treatment [5]. BD is characterized by episodes of extreme low mood (depression) and extreme high or irritable mood (mania or hypomania in its milder form). Challenging behaviors such as increased self-harm and suicidal behavior, excessive financial spending, sexual disinhibition, and heightened irritability can all escalate during mood episodes, and psychotic symptoms are also more likely to occur. Between episodes, functioning may return to normal levels, although many people do report problematic subsyndromal levels of depression, which impact on their functioning and relationships [6].

Both psychosis and BD present significant challenges to relatives, particularly in recognizing and understanding what is happening, living with the elevated risk of suicide, the impact on relationships within the family, and having to balance commitments such as caring and work.

First episodes usually appear in adolescents, at which point the individuals are still living with families [7]. In this study, we refer to the wider community of partners, friends, family, and caregivers as *relatives*, as this was deemed to be the most inclusive term without assuming the nature of the relationship. Relatives play a significant role at all stages of recovery; however, this unrecognized caregiving role can have adverse effects on relative’s psychological well-being, relationships, finances, employment, and quality of life [8-13]. Due to societal stigma related to mental health, relatives may struggle to seek help or to share their feelings and lived experiences with others [14]. This can result in isolation and loneliness and can influence their capacity to cope as well as to affect their own mental health [15].

The family unit affected by BD and psychosis-like experiences can benefit from information and educational support on how to support their relative [16]. Family intervention is used to involve all members of the family to discuss how such experiences affect the family unit and individual relationships. The aim of family interventions is to identify changes and strategies that could improve coping strategies of family members and establish a better family relationship. Family interventions require a trained member of staff to meet with family members face-to-face on a regular basis and have shown to be effective in reducing relapse rates for people with mental health problems [17,18] and improving the relative’s well-being [19]. However, delivering a family intervention through health and social care services can be challenging because of the (1) practical difficulties in gathering all family members in 1 room during working hours because of work and family commitments, (2) costly nature of face-to-face model, and (3) lack of resources in services [20]. The rates of implementation for family interventions in the United Kingdom vary from 0% to 53% [20], is up to 15% in Western Europe, and only about 10% of families receive family intervention in the United States [21].

### Relatives Education And Coping Toolkit Booklet (REACT)

To improve delivery of family intervention, we consulted relatives about the key challenges they faced and what kinds of support they most needed [22]. The outcomes of the consultation process informed the design of the Relatives Education And Coping Toolkit (REACT) booklet. The REACT booklet is an informative modular toolkit, which draws on key elements of family intervention, and can be used by relatives directly without the involvement of other family members or extensive service support. The REACT modules include information on managing symptoms, managing difficult behavior, coping with their own stress, information about medication, and understanding mental health services. The REACT booklet has shown to be effective during a randomized controlled trial (RCT; N=103) [23]. Those who had access to the REACT booklet in addition to usual treatment showed reduced distress, increased perceived support, and increased perceived ability to cope compared with controls with access to usual treatment only [24].

### Digital as a Vehicle for Providing Support

Despite the success of the printed REACT booklet, there are several limitations that the use of digital tools can address: (1) scalability both in terms of cost and access; (2) updating the information while health and social care services are constantly changing; and (3) booklets tend to be more generic and limited in scope. As we have progressed into the digital age, the provision of health and social care services has moved toward online models, which has, in turn, improved access for a wide variety of patient and caregiver groups [25-27]. Digital platforms that provide support for mental health have also proven to be successful and cost-effective [28-31] as they offer flexibility, accessibility, inclusivity, and anonymity, which can be appreciated in a stigmatized mental health context [32]. Therefore, we aimed to develop a Web-based version of the REACT booklet. In this paper, we describe a qualitative study that used a user-centered approach to design a widely accessible REACT Web-based intervention.

### User-Centered Approaches in the Design of Digital Health Interventions

Research suggests that effectiveness of Web-based health interventions correlates with users’ level of engagement [33-35]. However, attrition is a common problem across digital health technologies [36-38]. The importance of investigating approaches to improve user engagement has been highlighted in the literature [39], with the use of incentives [40] and prompts [41] identified as being methods of increasing engagement. The importance of taking users’ perception into account in the design of the content of Web-based interventions is needed to ensure (1) relevancy of content to their lived experiences, (2) consistency with their values, and (3) that the content provides additional benefit [42]. As such, the employment of user-centered methods, which aim to understand end users’ needs and values throughout the lifecycle of design, development, and evaluation, intended to improve user engagement [43]. Although the literature highlights the importance of user-centered design [44-49], using this approach in a mental health context can be challenging in terms of recruiting participants and understanding the lived experiences of those affected by mental health conditions [50,51].

In this paper, we present the results of our qualitative study. First, we conducted 2 workshop sessions with 24 relatives. These workshops aimed to understand (1) the current infrastructure (eg, the lived experiences of relatives and what support is currently available for them), (2) the barriers (eg, the main challenges for accessing existing support), and (3) the gap (eg, the support currently missing from the care system). This was followed by a workshop involving 2 relatives. This workshop aimed to verify and expand on the findings from the first 2 workshops and further explore the features that needed to be considered in the design of a supportive Web-based toolkit for relatives. Second, we conducted a small heuristic evaluation of a design prototype to better inform our final set of requirements. However, we focus the majority of our study on reporting the experience-driven needs and values that participants shared during our design process. The specific challenges faced by our participants, who were mainly older parental relatives of people with mental health problems, can be used as a starting point toward understanding how we might design inclusive and accessible digital interventions to support this complex user group. In this paper, the term *participants* is used when discussing relatives who took part in our study.

Through this study, we contribute (1) insight into the needs of relatives and their concerns about Web-based interventions intended to provide information and support, (2) a pragmatic example of a user-centered approach in designing a Web-based intervention, including the complexities of engaging a representative sample of full-time relatives in the discussion of highly sensitive topics, and (3) a set of design considerations for the development of a Web-based toolkit to support the informational and emotional needs of relatives.

## Methods

### Phase 1 Method: User-Centered Approach

In accordance with our ethics approval from Lancaster National Research Ethics Service Committee (15/NW/0732), participants were invited to take part in our study if they identified as a relative or close friend involved in supporting someone with BD or psychosis-like experiences. Our recruitment strategy involved advertising locally to obtain a convenience sample who would be able to attend face-to-face workshops. An email advertisement was circulated through the Lancaster University’s Spectrum Centre, which specializes in conducting research in BD and psychosis. Once participants indicated interest in taking part, an information sheet was sent by an email or post before attending the workshop to allow them to make an informed decision about attending and ask any questions in advance. Participants were offered a £20 Amazon voucher as an appreciation for their time and input. Each workshop was audio-recorded with participants’ permission and transcribed verbatim for later analysis (approach described below in the section Data Analysis). A total of 25 participants took part in this qualitative study. Although we aimed to recruit participants from a diverse demographic background in terms of age, gender, and relationship with the person with a mental health problem, the length of time being a relative, and computer literacy, the participants were predominantly female (18 females), over the age of 65 years (age range 21-75 years), parents (n=20), and infrequent computer users. Most participants had many years of experience of caring for their relatives (on average, approximately 10 years).

### Phase 1 Design Workshops

#### Workshops 1 and 2

We conducted 2 workshops with 13 participants in workshop 1 and 11 participants in workshop 2 (total n=24), which lasted for 2 hours each. Two researchers facilitated the workshops using a semistructured topic guide to lead the discussions and asking open questions to elicit a range of views. Our aim was to better understand the relatives’ needs, their context of use, and how they may, or may not, engage with online support in their caring role. First, the participants were encouraged to reflect and share their lived experiences as relatives of someone with a mental health problem, including how their relative was first diagnosed, how they became involved as relatives, the impact of mental health problems on their family unit and daily life, their current support-seeking practices, and any specific type of support or strategies they find most useful. Second, they were asked about the gaps in the support system for relatives, their views on how to overcome this gap, and their views on the role of online support. We then looked at the REACT booklets together and discussed the best ways in which these could be redesigned as a Web-based intervention and the types of support relatives would need to use this intervention.

#### Workshop 3

We conducted a third workshop with 2 participants to allow for an in-depth discussion around scoping the types of features that could be implemented on the REACT Web-based intervention. For this workshop, we aimed to bring together participants who were comfortable using computers. We also wanted to have an older and a younger representative to identify any possible age-related challenges relatives might have. The first participant (older adult, aged 61 years) had taken part in workshop 1 and so acted as a representative for the initial discussions, and the second participant (younger adult, aged 21 years) was new to the study.

Before the workshop, participants were given access to a website containing PDF information taken directly from the REACT booklet, which both the participants spent a considerable amount of time reading. During the workshop, participants were given brief information about the REACT project. First, participants were asked to highlight mental health–related and nonrelated webpages they liked or disliked, then we looked into their aesthetics and functionalities together. This was followed by visual demonstration of a series of prototypes, which represented design choices for the REACT Web-based intervention based on data from workshops 1 and 2. The prototypes were designed in conjunction with a Web design company and mainly focused on aesthetic aspects of the Web interface, that is, logo, font style and size, navigation menu, color scheme, and multimedia choices.

We wanted to know how to translate the values and needs participants highlighted in workshops 1 and 2 into functionalities that can be implemented in the REACT Web-based intervention. In particular, we wanted to know how to provide a positive user experience and, therefore, asked the participants to discuss (1) how to design the REACT Web-based intervention to be more engaging, (2) what features could motivate relatives to revisit the intervention, and (3) the types of support features, which can only be offered online. These design decisions were explored in depth in workshop 3, which focused on attempting to make clear design decisions to bring forward to the development phase for the REACT Web-based intervention.

### Data Analysis: Phase 1

The qualitative data collected from all 3 workshops were coded in NVivo 11 software (QSR International) [52] by 2 researchers using the Braun and Clarke [53] thematic analysis to identify key ideas to inform the design of the REACT Web-based intervention. The analysis was inductive and, therefore, data driven. Coders then worked together and discussed any discrepancies before agreeing on a final set of themes. Identified themes were discussed and refined with input from another researcher to ensure the themes were representative of the data and could inform the design of the Web-based version of REACT.

### Phase 2 Method: Heuristic Evaluation

The clinical effectiveness and cost-effectiveness of the REACT Web-based intervention is currently undergoing a national RCT [54]. We wanted to run a preliminary, surface-level usability evaluation of the REACT Web-based intervention with a small group of participants in preparation for the RCT. Our intention with this heuristic evaluation was not to evaluate the clinical effectiveness of the REACT Web-based intervention but to identify any inconsistencies, which could be addressed before the start of the RCT. We were looking for any inconsistencies in relation to language, functionalities, content, and structure of the intervention as well as whether the registration process is easy to follow and would make sense to the relatives. Using a similar approach to van der Krieke et al [55], we created a table of the questions that we were looking to address in this evaluation stage (see Table 1; questions adapted from the study by van der Krieke et al [55]). The questions were designed based on the 10 usability principles of Neilsen’s heuristic evaluation guideline [56]. All participants were informed about the aim of the evaluation and were instructed to think about our questions during their evaluation. To gain a better perspective of the typical users, we conducted this evaluation in 2 different settings of controlled and uncontrolled environments.

#### Controlled Setting Testing

This session was conducted in a controlled setting in a computing laboratory at Lancaster University. Overall, 3 participants took part; all were relatives and had mixed computer abilities. Two participants were new to the study. The one-to-one sessions were planned as *think-aloud* activities where participants were asked to walk through the intervention starting with the registration process, then using and testing functionalities, and documenting their experiences as they went through. The participants were asked to talk about their experience as they were using the intervention and report any incidents related to the questions in Table 1. Each session was audio-recorded and was used as a feedback to evaluate and refine the system requirements. Participants were provided with the opportunity to get in touch via email after the session to provide any further thoughts or ideas they wished to be included in the final development stage.

#### Uncontrolled Field-Testing

Because relatives are more likely to access the intervention from their own personal environment and without any assistance, it is important to review the intervention in an uncontrolled setting. A total of 8 participants were given access to the REACT Web-based intervention for 2 weeks, were asked to use it from their preferred environment (eg, their home), and provide feedback via email. Participants were recruited from our service user researcher group, which is a group comprising people with mental health problems and their relatives.

**Table 1:** Questions adapted from the study by van der Krieke et al to examine the usability of the Relatives Education And Coping Toolkit (REACT) Web-based intervention.

Usability principle	Question
Visibility of system status	Are the steps in the registration process clear to relatives?
	Is the intervention unresponsive or slow at any point?
Match between system and the real world	Does this intervention represent real-world experiences of relatives’ population and whether or not speaks in a language or uses terminologies that are familiar to this group?
User control and freedom	Is it clear for relatives which actions or activities are private and public?
	Is it clear that user can make the decision on how to engage with the intervention (unrestrictive model)?
Consistency and standards	Are the content, terminologies, and features of the intervention consistent throughout the intervention?
Error prevention	What mistakes are likely to occur during the registration process and completion of the eligibility questionnaires?
	Does the intervention have capacity to prevent or act on these mistakes?
Recognition rather than recall	Are the list of available functionalities, icons, and structure of the intervention clearly explained to the relative at all time?
Flexibility and efficiency of use	Does the relative have control over how they wish to use the intervention (personalized manner)?
Aesthetic and minimalist design	Are features of the intervention easy to understand, distinguish, and use?
Help users recognize, diagnose, and recover from errors	Is there enough clear instruction on how to get in touch and report any issues to the team?
Help and documentation	Is there enough guidance on how to use the intervention?

### Data Analysis: Phase 2

The qualitative data collected from the controlled setting testing were analyzed by 1 researcher using content analysis [57] to identify the improvements suggested by our participants. Participants who took part in the uncontrolled field testing provided their feedback by email, which consisted of mainly bullet points of recommended changes. The list of all identified changes from both settings was collated by 1 researcher, which was then passed to our internal software team to be addressed.

## Results

### Phase 1 Findings

Although the themes and subthemes are described as discrete, themes are to a large extent intertwined, building, or expanding on previous themes. A total of 9 themes emerged from all 3 workshops’ data, which were then synthesized into the 3 wider theme headings that we used to illustrate our findings. The 3 main identified overarching themes are (1) caring as an invisible role, (2) support needed for relatives, and (3) concerns about online support. Although relatives’ experiences as caregivers have been widely researched and described elsewhere [58-60], it is important to provide a context for the experiences of those involved in our study. We first report these broad themes to provide an insight into the themes of discussion. We then move to discuss the design requirements for the REACT Web-based intervention, which was generated from workshop 3. We append the quotes along with both workshop and participant number to distinguish sources of data (eg, a quote from workshop 1 participant 9 would be [WS1, P9]). In workshop 3, P2 represents the younger adult participant.

### Caring as an Invisible Role

#### A Change in Identity

Participants reflected on their personal experiences around the process of becoming a relative of someone with a mental health problem. Participants talked about feeling a loss of sense of self in their journey of becoming a carer. They felt they had been pushed into a *carer* role and found it hard to maintain their identity as a mother, brother, and so on. The role of the carer was particularly unappealing as there was no formal training or guidance for this role; so, as well as being enforced, it was also very challenging:

I don’t want to be a carer, I don’t like the word. I’m a Mother.

WS1, P9

Many participants described how it took years for them to learn strategies to cope with the impact of mental health problems on the rest of the family, including (1) how to cope with new responsibilities and burden of care, (2) the changes the caregiving role brings to relative’s personal and professional life, (3) coping with emotional side effects, and (4) societal stigma surrounding mental health.

#### Impact on the Whole Family

Participants described the very broad impact of mental health on the wider family and the need for support for all family members:

Our youngest son didn’t understand what his brother was going through. He may have known about his brother thinking that people were after him, but it must have been terrible for him and in fact not long ago he actually left the family.

WS1, P1

In addition, participants discussed requiring support not just as relatives but also as individuals in their own right, with other responsibilities in their lives:

I might have had a right morning with my son; threatening suicide or wrestling for my own. And I’d have to go in work...change into my uniform and drive to work and I’ve got a lump in my throat. And I’d phone my partner [saying] I’m going to cry.

WS2, P3

#### Social Isolation

Social interaction is challenging for relatives of those with physical illness as they may have little respite because of the need for constant caring. However, with mental health, social isolation can be further exacerbated by the result of stigma and lack of public awareness about mental health. Participants talked about having found it difficult to open up and discuss their mental health–related experiences with others. Finding other people with similar lived experiences had been initially challenging, but once accessed, became invaluable, not only for emotional support but also for signposting to important information and guidance elsewhere. Many were part of charity-run face-to-face peer support groups facilitated by an expert relative:

The person who runs our small group is a God send, what’s worrying is if she couldn’t do that job what would we do with it? That’s what I always think about because there’s got to be a system there that does what she does...

WS1, P4

Although valuable, these groups were considered to be scarce and almost invisible to newly adopted relatives.

### Support Needed for Relatives

#### Informational Support

Participants acknowledged that it can take years for relatives to learn about the mental health problems and, on reflection, identified 3 types of educational information sources that could be useful for other relatives:

1. All available sources of support (eg, local support groups and national charity organizations):
  This exists. That exists. You can read this. You can read that. You can go here. You can go there. You’ve got a right to this—this is practical help—not the general pat you on the back and say everything’s alright and happy clappy, and let’s be friends. But actual hard practical, meaningful.
  WS1, P6
2. Information about medication, including types, side effects, and how to manage doses:
  We’re never given sort of like a comparative— information about the various anti-psychotics. They had awful side effects.
  WS2, P5
3. Legal rights:
  I think actually what carers are entitled to under the law is very different from what they get in real life. And you’ve got to know.
  WS1, P1

Overall, participants agreed that knowledge is power and that relatives of those who have been newly diagnosed would benefit from guidance on how to get help and to be signposted to trusted and up-to-date resources.

One of the challenges of a self-management toolkit is that there are often no right or wrong answers. Participants highlighted the importance of vicarious learning in addition to didactic instruction. One participant felt that a limitation of many of online support they had reviewed was that they tried to provide checklists of what to do:

like a lot of websites will say, oh why don’t you try meditation, going for a walk.

WS3, P2

Instead, it was suggested that online support needs to be thought provoking; it needs to facilitate *thinking and reflecting* exercises that enable relatives to learn problem-solving strategies that can then be applied to their own particular context:

...like stuff that helps you ask questions and helps you think about what you’re feeling rather than like, try this, because there’s only like so much a hot bath can cure.

WS3, P2

Participants suggested more emphasis should be placed on inviting relatives to dip in and out, in an order and frequency of their choosing, without the need to complete the intervention in a sequential order:

Normally websites like this are just information, read it and then go. But because this is more of a programme that you can work through. So I’d have to understand that’s what it was for and that was how I’d used it in a really good clear way.

WS3, P2

#### Emotional Support

Participants talked about the emotional impact of supporting someone with psychosis and the importance of emotional and practical support:

I had some professional experience [in mental health] but it’s completely different when you are emotionally involved in somebody. And it’s literally like somebody’s just parachuted you into a foreign country. You’ve no idea of what should be happening, what is available. And you need to know that sometimes to be able to get it. But the peer support is about emotional support. And I think what health professionals sometimes don’t understand is by the time you get to them you’ve been doing this for months. Twenty four hours a day, seven days a week. And the emotional toll on you.

WS2, P2

They talked about the importance of hearing that their experiences were not unique, the need for explicit reassurances that the development of mental health problems within the family was not their fault, and that they were doing all they could to manage the situation:

My mum never forgets this nurse who said to her, it could happen to anybody. This is not your fault. You’re doing everything you can. And that just lifted that guilt off my parents.

WS2, P7

Opportunities to have social contact with similar relatives to share lived experiences and feel connected and supported were particularly valued:

But there’s nothing better than seeing that somebody else has had the same fears and guilt to start with. Worries about the future and practical travel problems, just to hear that other people have got the same issues that you have.

WS3, P1

Participants explained that for some questions, there is a lack of informative answers on Google:

How much am I supposed to do? How strict am I supposed to be? When’s the point when I back off? And that’s not really a question that I felt I could ask Google cause I’m not going to get anything useful from that.

WS3, P2

Instead, relatives are looking for someone to talk to even if they realize that there is nothing they can do to resolve the situation; at times knowing that they are not the only person debating the issue could serve to help them cope better.

#### A Recovery-Focused Approach

Although most of the discussion revolved around challenges relatives faced, the need to focus on positive outcomes was also important to relatives. One participant explained how she desperately struggled in finding positive role models for her son:

But then I realised that the positive role models don’t want to go back and look again. And there’s got to be thousands of recovered or people who are managing their condition but they don’t really want to join the club. And that would be priceless to have more positive role models. People who have managed and are managing their conditions or have completely recovered.

WS3, P1

There was a general feeling that both relatives and people with mental health problems would benefit from hearing positive stories to give them hope that recovery is possible.

Participants expressed that supporting someone with a mental health condition is often an ongoing journey and suggested to offer a personal space in which relatives can save useful information to revisit easily in the future:

Like some kind of like scrapbook section. Not called that but that kind of thing where like people just put different stuff in.

WS3, P2

The value of this was not only in having a useful place to store things to be revisited but also to facilitate a process of reflection on progress over time:

...it’s very affirming to go back to some of the earlier learning content to realise that you have learnt, you know, I’ve acted correctly. You have been a good carer.

WS3, P1

### Concerns About Online Support

#### Questioning the Confidentiality of Online Support

Participants debated on the use of Web resources in a mental health context and expressed that online support can be seen as a "big scary virtual world" for some relatives. Some participants expressed fears toward online activity, and many felt reluctant to input personal information to any website. Their fears were that once shared, information posted online could never be removed and will always be “Googleable.” There was also an ethical dilemma that, in sharing their own experiences online, they may be sharing their relatives’ personal experiences without their explicit consent. Participants agreed that it is often unclear as "whose story is it” that they might be sharing online:

I don’t mind saying anything about my own medical symptoms or if I had a mental health problem but I don’t feel comfortable about using it about my relatives.No, but on the other hand if my anxiety and stress and my needs are because myrelative’s issues aren’t being addressed.Then it is about me but it’s still about her. So I wouldn’t feel comfortable putting anything down there. But everybody’s different so I think there needs to be a number of ways to access this information.

WS1, P10

#### Wider Impact of Being Active Online

They described several practical challenges about using the internet, including limited access and skills:

I live in the country and my internet doesn’t work half the time and my computer is probably my biggest source of stress.

WS1, P10

However, it was not only practical issues that made them concerned. This dilemma was exacerbated by the fear of the impact their posts might have on their relative if they saw them:

I feel uncomfortable about discussing [my relative online] if my relative happened to get access to it, it could trigger a major episode.

WS1, P8

Many reported that receiving destructive comments on social media could place significant strains on relatives:

You might just get some people who are just time wasters. You might get some destructive things.

WS2, P1

However, they acknowledged the need for moderators:

Usually though you have moderators on sites like that.

WS2, P6

Participants acknowledged that to eliminate stigma, mental health needs to be discussed openly but feared this could cause more damage:

But I’ve always feared letting [my relative] know how I’m feeling about things...I’m not sure how far we can open up...

WS1, P5

In discussions around prompts, participants felt that options should be made available for people to customize the frequency and mode of delivery of prompts. They disliked receiving too many prompts and expressed that if they received prompts too often they would feel “suffocated” or “pressured,” especially if they are having a good day and, therefore, “reject” and “unsubscribe.” They also preferred receiving person-centered prompts:

What’s your question this week? What are you worrying about this week? You never get any e-mails that just say how are you doing? How are you

WS3, P2

#### Legitimacy of Online Support

Simplicity, easy navigation, and professional look were all seen as features that would attract relatives by assuring them that this intervention is legitimate:

...cause you look at it and you go, oh wow, this looks legitimate like I can trust this. And then you start building up that trust and start using it.

WS3, P2

Participants compared the look of a website with a building and suggested not to aim for a slick look, such as a building with shiny floors that feels corporate, instead, something professional that provides the information but looks simple. Overall, the design of the toolkit was felt to be very important in engendering a sense of trust in participants. Participants emphasized on the importance of privacy and security and that they need to be assured that the online support is a safe environment for sharing stories and experiences:

It would have to be very secure the site, because who can access this site, I mean I don’t go on Facebook or anything like that because I just think that a lot of it is not secure. Anybody can [access], you know.

WS2, P1

Overall, participants had mixed views about the value of online support but felt that people in the next generation may be more positive:

We’re all of a certain age. And what I’m finding is that, the people in the group who are a lot younger are actually perfectly happy to go all over Facebook.

WS1, P1

And may even be put off by paper-based support:

But it’s often a generational thing and a lot of younger carers are siblings. Would never dream of getting something off paper they would automatically go online.

WS2, P6

### Relatives Education And Coping Toolkit Web-Based Intervention Design

**Figure 1:**
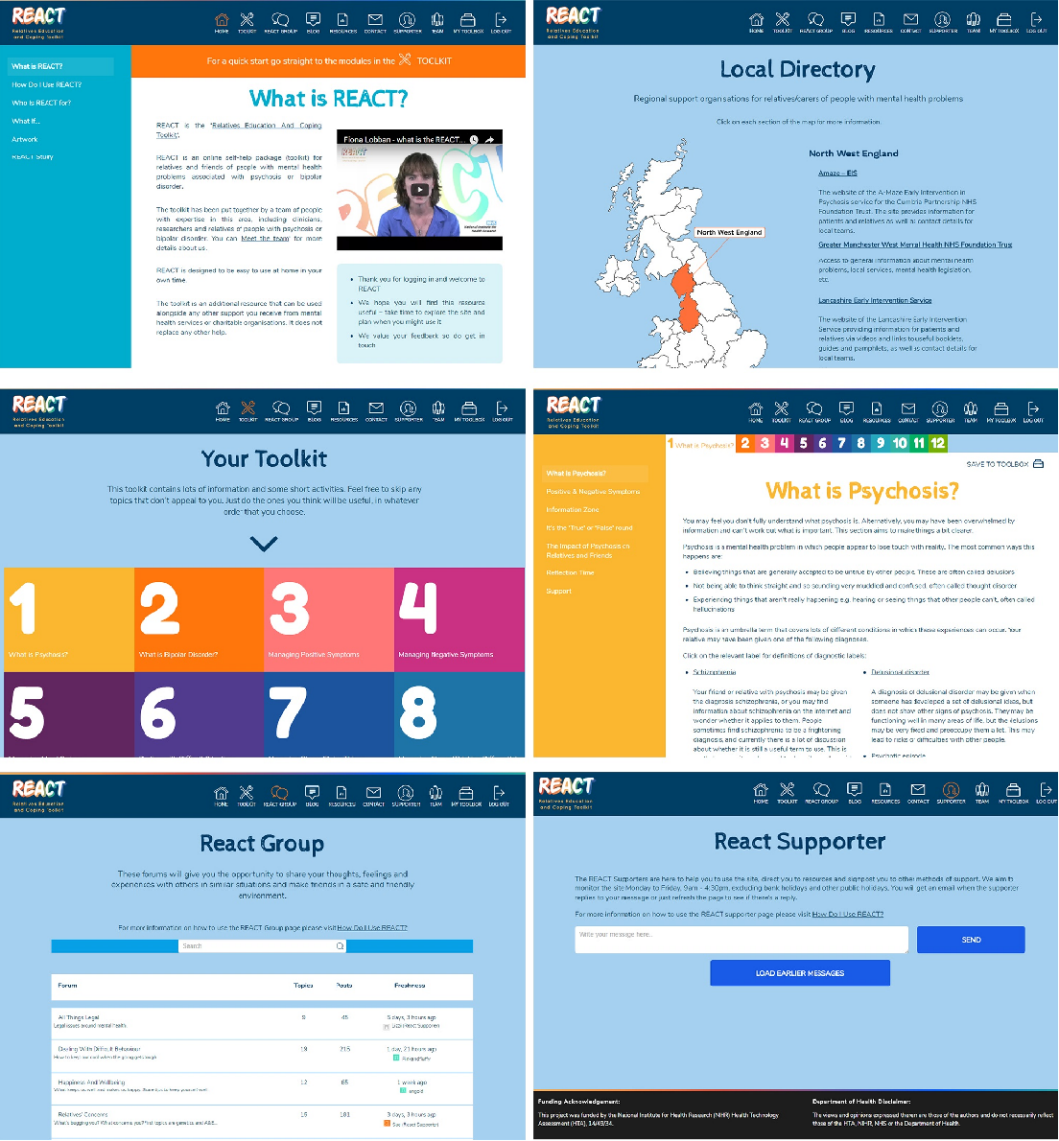
Screenshots to show features of the Relatives Education And Coping Toolkit (REACT) Web-based intervention.

The REACT Web-based intervention was developed using WordPress [61]—an open source Content Management System—and can be accessed via a weblink to sign up. However, the weblink is currently not openly available as REACT’s clinical effectiveness is still being tested under controlled conditions. The interface is responsive to various devices, including mobile phones, tablets, and desktop. The REACT Web-based intervention (Figure 1) is composed of 6 features: educational modules (REACT), list of national and local support resources (resource directory), discussion forum (REACT group), moderators (REACT supporters), personal space (my toolbox), and a blog section.

The REACT Toolkit was designed based on the REACT booklet and features 12 modules covering various aspects of mental health. Each module contains information, self-reflection activities, and short video clips of clinicians, REACT supporters, and lived experiences of relatives (who are played by actors). Further details of the modules can be found in the study by Lobban et al [54].

To support participants’ concerns around finding reliable and trusted information online, we created the resources directory feature, which aims to bring together an extensive list of Web resources that relatives may find useful at all stages of caring. The resource directory offers more detailed explanations and information on various topics relevant to supporting someone with a mental health problem. This feature has 4 main categories, each covering a range of different topics: (1) national organizations supporting relatives, that is, charity organizations; (2) government guidance, that is, the department of work and pension; (3) topic-specific information, that is, insurance and driving license;and (4) local resources to support relatives of people with mental health problems—relatives would access the UK map and by selecting the area they live in, they will be provided with a list of support available in their area.

In response to participants desire to be emotionally supported and linked to others with similar experiences, we created the REACT group—a moderated space for sharing knowledge and emotional peer support. The REACT supporters who are trained relatives with lived experience of caring for a family member moderate the REACT group are available for private questions. They also update the resource directory on a regular basis.

My Toolbox is a private space to store the information that is important to the relative. My Toolbox was created in response to participants’ desire for a personal approach to be able to save interesting content and the ability to revisit in a less time-consuming and less stressful manner, particularly during crises. Throughout the intervention, relatives will see the *save to toolbox* symbol, which once clicked allows the page to become stored in their My Toolbox space. The blog facilitates an opportunity to update relatives of the latest news or to provide a topic-relevant story of the most sought-after topics on the REACT group by invited guests, that is, clinicians, researchers, or our REACT supporters.

### Phase 2: Findings

We evaluated the initial system requirements against the feedback from both heuristic evaluations, and where possible, the findings are related to the Neilsen’s guideline [57]. Any identified changes were addressed by our internal software team. The majority of reported issues were relevant to Neilsen’s Consistency and Standards principle, which was focused on typos within the text, rewording some sentences, and resizing images. There were also some instances in which some features were unresponsive, for example, a video would not play or a link would not redirect, which were related to the Visibility of System Status principle. Some participants who conducted the evaluation from home reported that initially they found it difficult to understand how to use the intervention, that is, the role of My Toolbox was unclear; this corresponds to the aesthetic and minimalist design principle. Furthermore, related to the Match between System and the Real World principle, they recommended that using more images of the REACT supporters’ faces throughout the intervention would add value in terms of getting a sense of the real people behind the intervention and promoting relatability and trust. In response to the 2 former comments, we created additional short videos of the REACT supporters introducing themselves, explaining the functionalities of the REACT Web-based intervention, and providing instructions on how to navigate and use the intervention. With regard to the content of the REACT and the Match between System and the Real World principle, participants found the topics covered relevant and useful:

...this is really useful information that I was never made fully aware of when we needed it.

A number of participants highlighted a further issue with respect to the Match between System and the Real World principle, and they expressed how they disliked acronyms as they find them difficult to understand. They suggested to list both the acronyms and their full names as a “jargon buster” type approach, which was added to our resource directory. Related to the Visibility of System status principle, participants commented that they could watch the lived experience videos using their home internet network and found them useful to get a sense of *“* someone else understands what this is like” and to give hope to relatives when they are struggling or are in a crisis. This confirmed that the intervention is representing real-world experiences of relatives related to the Match between System and the Real World principle. Participants commented positively on the REACT group and found it easy to use:

I really like this idea of a virtual meeting place for sharing experiences and getting support, I think this might prove to be one of the most helpful areas of the site.

## Discussion

### Principal Findings

The aim of this study was to develop an accessible Web-based intervention to support the informational and emotional needs of relatives of people experiencing psychosis or BD. We used a user-centered design approach to better understand the needs and values of relatives. Our findings showed that relatives preferred technologies that (1) they can place their trust in, particularly when discussing a highly sensitive topic, (2) enable learning from the lived experiences of others while retaining confidentiality, and (3) they can work through in their own pace in a personalized manner. These findings have clear implications for the design of our Web-based version of REACT and for others developing similar Web-based interventions for people affected by mental health conditions. Our discussion is organized into the following design considerations: (1) designing to engender trust and (2) designing for relatives with many roles.

#### Designing to Engender Trust

As Briggs et al [62] note, our findings show that it is not simply the provision of health-related information that is important, it is the establishment of trust and credibility. There was much discussion about the need for trust in information sources in the sense that they are reliable and legitimate. The guideline for designing trust into online experiences [63] suggests making it easier for users to access the enforced privacy policies to establish trust. As noted in the study by Sillence et al [64], trust is closely associated with risk, and in the context of designing a toolkit for relatives, this goes beyond the risks surrounding self-disclosure and is more concerned about revealing information about someone else’s experiences. Relatives discussing their experiences of caring for someone with a mental health condition could be seen to give away personal information, including the identity of the person with a mental health problem. Although health is perceived as a high-risk domain for seeking support online [65], mental health faces additional challenges establishing trust with online users because of the stigma related to mental health conditions. As such, factors affecting trust in mental health need to be understood if the online support is to be valued in long-term use.

It was apparent that our participants spent a number of years reaching a point in which they understood the mental health condition enough to reach out to face-to-face support groups and to gain an understanding of the care system infrastructure. Reaching this point is a lengthy and tiresome journey, and although it feels as if they need to be part of a group to know their rights, peer support groups are small and almost invisible to relatives who are new in their caring role and may be feeling overwhelmed adapting into this role. Our findings highlighted that although relatives had difficulties finding and accessing support, privacy and security concerns may inhibit them from accessing this support online. Although they valued being connected to others with similar experiences, they raised concerns about the misuse of discussion forums and emphasized the need for moderators. Our participants wanted to know that their personal information and discussions will be kept confidential, particularly when discussing highly stigmatizing topics such as mental health. For the majority of participants, finding a way to share lived experiences while maintaining the confidentiality of oneself and the person they care for was an important value, and they would like to access tools that would support them. As such, we encourage future researchers wishing to work within this space to make it clear what happens to individuals’ data, explain what safeguarding measures are in place to protect the privacy of individuals, and clarify how the provision of a safe environment for interacting with others has been embedded within the core of the design (eg, providing moderators and setting ground rules for interaction).

In addition, our participants wanted to know that the information provided in the toolkit was *credible* and that real people with clinical expertise and lived experiences of mental health problems had been engaged in its development. Aesthetics, branding, quality of information, and relevancy of the information to each individual user were all seen to influence credibility. This is consistent with findings in the broader literature around trust in health information [64,66,67] and highlights the necessity of engaging multiple members of health community in the design of tools aimed at targeting information provision and support needs.

#### Designing for Relatives With Many Roles

One of the continuing concerns participants expressed was the need for retaining a sense of ownership during care, particularly when they feel that the caring role has overtaken other life roles (eg, being a partner, parent, or sibling). We suggest that we do not just focus on designing for *the relative* as a user but also explore designing for relatives with other roles and responsibilities (eg, relatives managing a career and relatives with young children). We need to make sure that we develop online support that allows relatives to retain their identity rather than just focusing on caring. This is reflective of previous work, which has explored the design of technologies that fit into the messy daily lives of relatives [68,69]. Similarly, online support tools should be designed in such a way to let relatives work at own pace while being able to carry on with other aspects of their life. It was apparent in our study that relatives are not homogenous in their experiences and, thus, their priorities and their engagement with online support, therefore, having space to tailor and personalize engagement is essential.

Monk et al addressed the need to move from designing for usability to enjoyment [70], which is also echoed in feedback from our participants. Our findings indicate that relatives require technologies that take away the pressure and stress resulting from caring duties, and they preferred informational support that provides a positive, although realistic, representation of mental health problems. Our participants wanted to ensure that we provided a message that recovery is possible, that relatives will eventually identify their own coping strategies, and that the future can be bright. We tried to convey these positive messages throughout the intervention as well as the video stories of lived experiences of relatives. The assumption is that with mental health, we deal with very serious challenges, and when it comes to design of software systems, we only focus on usefulness rather than a system that can be both useful and motivating. Hence, from a design perspective, we still need to investigate positive approaches and how to promote positive emotion in this very complex and challenging domain of mental health.

### Strengths and Limitations

The main strength of this study is the employment of user-centered design methodology where in-depth perspectives of relatives of people with psychosis or BD were collected. This early involvement in the design and heuristic evaluation process of the REACT Web-based intervention can increase the likelihood of developing a useful and trusted supportive toolkit. The main weakness of the study is the use of a convenience sample that was skewed toward older females aged over 65 years, with limited computer literacy. The sample was recruited through existing links and local carer groups and is, therefore, not representative of relatives supporting people with mental health problems or those likely to engage with Web-based interventions.

Despite not intending to recruit an older sample of participants, this proved beneficial. First, we consulted experienced relatives who had been providing support for their family member for a number of years and, therefore, had expert knowledge in the challenges, solutions, and support that families of people with newly diagnosed mental health problems might need. In addition, our participants had less familiarity with online support, which resulted in them sharing important concerns related to privacy, confidentiality, and trust, which may not have been shared by younger and more *tech-* *savvy* participants who are used to modern social media. Although initially we considered our largely older sample as a limitation to our study, we found it particularly helpful to hear the concerns of these relatives as it made us think about how to address these in our design. This resulted in more inclusive design decisions and opened up our toolkit to be accessible for a wider range of users.

### Conclusions

Through this study, we have offered a deepened understanding of the specific needs and values of relatives of people with experiences of BD or psychosis to inform the design of a Web-based intervention to support them in their caring role. Although it is challenging to meet the needs of all end users, engaging them in the development of resources to support them is valuable and necessary. Our study has highlighted the challenges relatives face during the process of becoming caregivers and the need for providing a trustworthy, supportive tool where they can gain informational and emotional support and gain access to others with similar experiences. Our heuristic evaluation of the REACT Web-based intervention showed promise in terms of perceived usability by a small number of participants. An RCT is currently underway to evaluate its clinical effectiveness.

## Abbreviations

BD: bipolar disorder
RCT: randomized controlled trial
REACT: Relatives Education And Coping Toolkit
